# Letter to the editor on “the implementation research logic model: a method for planning, executing, reporting, and synthesizing implementation projects” (Smith JD, Li DH, Rafferty MR. the implementation research logic model: a method for planning, executing, reporting, and synthesizing implementation projects. Implement Sci. 2020;15 (1):84. Doi:10.1186/s13012-020-01041-8)

**DOI:** 10.1186/s13012-021-01169-1

**Published:** 2021-11-17

**Authors:** Anne E. Sales, Douglas P. Barnaby, Victor Cattani Rentes

**Affiliations:** 1 Sinclair School of Nursing and Department of Family and Community Medicine, University of Missouri, Columbia, MO USA; 2 Department of Learning Health Sciences, University of Michigan, Ann Arbor, MI USA; 3 VA Center for Clinical Management Research, VA Ann Arbor Healthcare System, Ann Arbor, MI USA; 4 Northwell Health Feinstein Institutes for Health Research, Manhasset, NY USA

We write in response to the paper published by J.D. Smith and colleagues titled “The Implementation Research Logic Model” [1]. In that paper, Smith and colleagues describe an innovative logic model whose core purpose is to specify the relationships between determinants, strategies, and outcomes in an implementation project and provides an explicit description of the mechanism(s) linking selected strategies and outcomes. This chain of relationships helps support selection of strategies, a critical step described in many process models guiding implementation practice, and implementation research. The form of logic model described in their paper, and the methods they describe to use this model, represents important progress in this difficult and complicated area of systematic implementation.

A central premise of their paper is that inadequate specification of implementation strategies leads to obfuscation in “identifying the factors responsible for successful implementation and prevents learning from what contributed to failed implementation.” We agree—but note that this is precisely the function of “determinants” frameworks in Nilsen’s seminal 2015 paper [2]. The inductive approach used to develop the “Implementation Research Logic Model” yielded a very helpful tool, but one which is perhaps still not fully developed, and may not provide enough guidance to move the field forward to the essential point of how to link between determinants that have been assessed as extant and important, and strategies to overcome (in the case of negative determinants or barriers), or enhance them (in the case of positive determinants or facilitators).

The possible missing link may be the *causal mechanisms of the determinants*, which provides the same underlying theory logic for the determinants. The theories of change referred to in Smith et al. and demonstrated in Lewis et al. [3] require a theory-derived understanding of what causes a determinant to exist in the first place, which then logically should link to a strategy that will be effective in overcoming it; Smith and colleagues allude to this but do not include determinant causal mechanisms in their models. As proposed, and demonstrated, in the Smith et al. paper, a logical leap is still required between assessing a determinant and proposing a strategy. This can be filled through brainstorming, or other approaches, and a wide variety of stakeholders (researchers, clinicians, community members) can participate. A set of unstated causal mechanisms underlying the assessed determinants is likely to be implicit in these approaches. We argue that it is essential to make both the causal mechanisms of the determinants and the mechanisms of action of the strategies, which are included in the Smith et al. paper.

This discussion points out an important gap in our existing literature. As yet, despite the large number of existing frameworks in implementation and/or dissemination sciences [4, 5], none explicitly nominate either causal mechanisms for determinants or the mechanisms of action for strategies.

We believe that further development of the science of implementation requires, urgently, that mechanisms of action for all implementation strategies be proposed, disseminated, and discussed, those already cataloged in existing frameworks (such as in the ERIC project [6]).

As a corollary, existing and future determinants cataloged in frameworks need to include proposed causal mechanisms. We would add to the short list of determinants cited by Smith et al. another, more recent consolidated checklist or framework, which incorporates key elements of both of these older frameworks, the Tailored Implementation for Chronic Diseases checklist or framework [7]. Derived from systematic review of 12 prior determinants models or frameworks, this consolidation provides an excellent starting point for developing causal mechanisms for determinants.

We applaud the insights in the Smith et al. paper. Our proposed changes simply extend their important work by adding important elements to address the problem of how to use existing knowledge to select implementation strategies, a long-standing area of confusion and complexity in understanding the practice, and the science, of implementation.

## Data Availability

Not applicable.
